# Gridded, high-resolution ocean observatories initiative profiler data from the Washington continental slope, 2014–2025

**DOI:** 10.1016/j.dib.2025.111861

**Published:** 2025-07-08

**Authors:** Craig M. Risien, Russell A. Desiderio, Jonathan P. Fram, Edward P. Dever

**Affiliations:** College of Earth, Ocean, and Atmospheric Sciences, Oregon State University, Corvallis, OR 97331, USA

**Keywords:** Seawater temperature, Practical salinity, Potential density, Dissolved oxygen, Climatology, California current, Mclane moored profiler

## Abstract

The NSF Ocean Observatories Initiative (OOI) Coastal Endurance Washington Offshore Profiler Mooring (CE09OSPM) was first deployed in April 2014. The mooring is located on the Washington continental slope about 60 km west of Grays Harbor, WA at 46.8517°N, 124.982°W. This mooring includes a McLane® Moored Profiler (MMP), which carries energy-efficient instruments that simultaneously measure water temperature, conductivity, pressure, and dissolved oxygen, as well as photosynthetically active radiation, chlorophyll-a fluorescence, coloured dissolved organic matter, optical backscatter, and water velocity. Moving at about 25 cm/s, the MMP collects up to eight profiles per day between approximately 35 m and 510 m water depth. This data article describes a data set that consists of 3244 daily averaged temperature, practical salinity, potential density, and dissolved oxygen profiles collected between October 2014 and May 2025 that were processed using a MATLAB® toolbox that was specifically created to process OOI MMP data. The toolbox imports unpacked MMP data files, applies the necessary calibration coefficients and data corrections, including adjusting for thermal-lag, flow, and sensor time constant effects, and produces a final, 0.5-dbar binned data set. From the daily, gridded profiler data, we calculated seasonal cycles for each variable using a least squares fit of the annual, semi-annual, and triannual harmonics. These gridded profiler data, which are vital for advancing our understanding of subsurface oceanographic phenomena — including modulation of the California Undercurrent, water mass and upwelling source water variability, marine heat waves, ocean acidification, and the increasing prevalence and severity of seasonal hypoxia in the Northern California Upwelling System — are available via Zenodo at https://doi.org/10.5281/zenodo.15627742.

Specifications Table

**Table:** 

Subject	Earth & Environmental Sciences
Specific subject area	Hydrographic profiler data collected at 46.8517°N, 124.982°W
Type of data	Figure Raw, Processed
Data collection	This data collection consists of >24,000 individual Conductivity-Temperature-Depth (CTD) and dissolved oxygen profiles that were averaged to form a data set of 3244 daily mean profiles. These data were collected from October 2014 to May 2025 at 46.8517°N, 124.982°W using a McLane® Moored Profiler (MMP). The MMP, moving vertically at about 25 cm/s along a plastic jacketed wire rope, collects up to eight CTD and dissolved oxygen profiles daily using a Sea-Bird Scientific 52-MP (SBE 52-MP) CTD instrument and an associated Sea-Bird Scientific (SBE 43F) dissolved oxygen sensor. All four sensors (temperature, conductivity, pressure, and dissolved oxygen) sample at 1 Hz.
Data source location	Data were collected by the NSF Ocean Observatories Initiative Washington Offshore Profiler Mooring (CE09OSPM) located at 46.8517°N, 124.982°W between approximately 35 and 510 m water depth.
Data accessibility	Repository name: Zenodo Data identification number: 10.5281/zenodo.15627742 Direct dataset link: https://doi.org/10.5281/zenodo.15627742 Zenodo is an open repository operated by the European Organization for Nuclear Research, known as CERN.

## 1 Value of the Data

• Using the 10-year time series presented here, researchers can analyze and characterize upwelling source water variability at intra and interannual time scales. Such variability is an important component of coastal ocean acidification and hypoxia in the California Current System [1,2].
• This data set provides valuable information on the formation and evolution of subsurface marine heat waves in the northeast Pacific Ocean [3].
• These data can be assimilated into reanalysis and state estimate models including GLORYS (Global Ocean Reanalysis and Simulations [4]) and ECCO (Estimating the Ocean Circulation and Climate [5]) to improve the accuracy of subsurface fields.
• The data set can be used to study seasonal and interannual variability of the California Undercurrent [6,7].

## 2 Background

While the decade long time series described here is provided to the research community at https://thredds.dataexplorer.oceanobservatories.org/thredds/catalog/catalog.html, the NSF Ocean Observatories Initiative (OOI) does not provide gridded McLane® Moored Profiler (MMP [8]) data that have been adjusted for thermal-lag, flow, and sensor time constant effects. Using the *mmp_toolbox* described below, we have produced a quality-controlled data set, which includes all the necessary corrections and that has been consistently gridded in space and time thus making the data more available and accessible to a broader swath of the marine research community. This toolbox was developed such that it can be used to process other OOI MMP data sets that have been collected by the Pioneer Array, originally located on the New-England shelf and now located in the Mid Atlantic Bight off North Carolina, as well as the high-latitude OOI arrays located at Station Papa, in the Irminger Sea, in the Southern Ocean southwest of Chile, and in the Argentine Basin [9].

## 3 Data Description

**Fig. 1:**
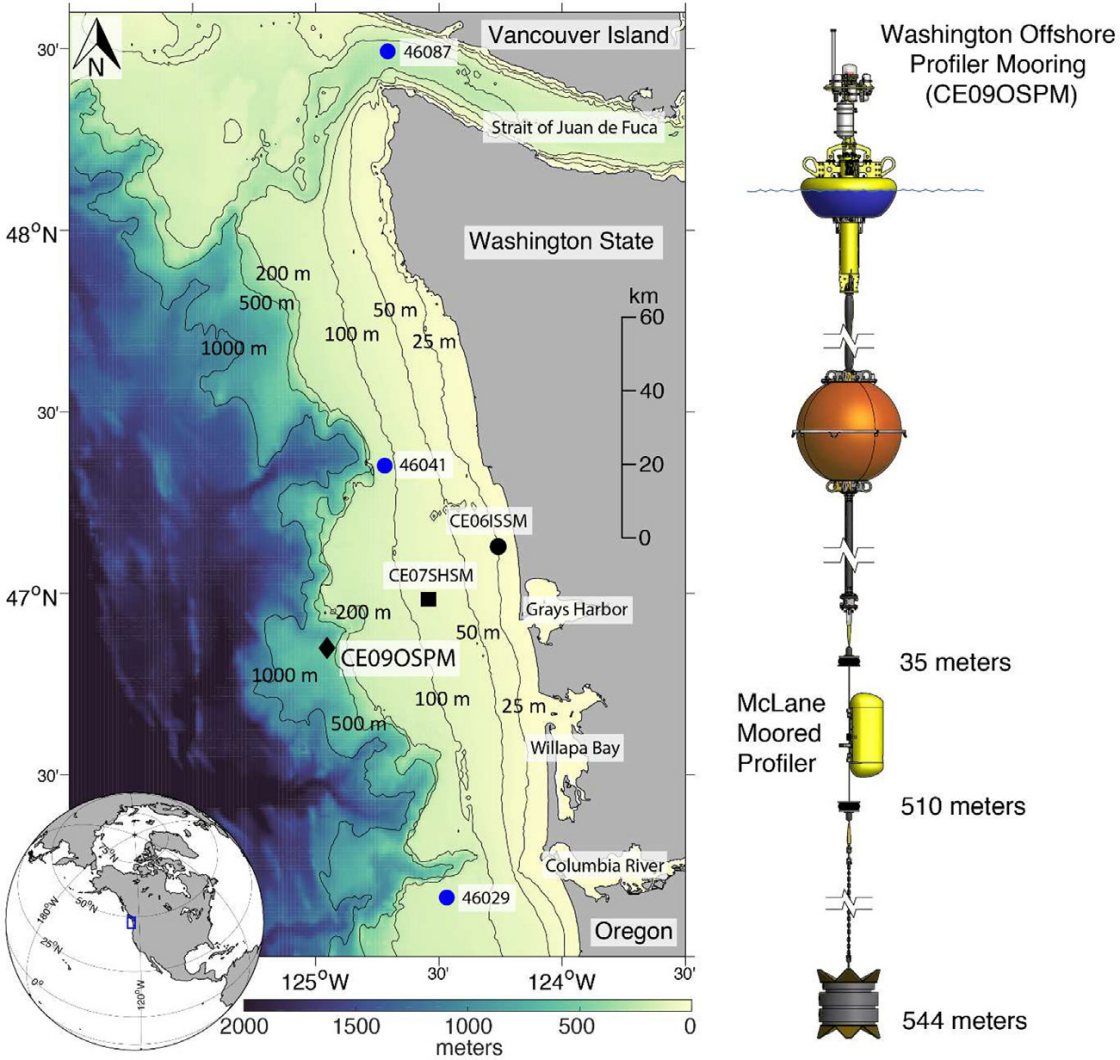
(Left) A map showing GEBCO bathymetry [10] including the 25, 50, 100, 200, 500, and 1000-meter isobaths, the locations of the OOI Washington Inshore (CE06ISSM; black circle), Shelf (CE07SHSM; black square), and Washington Offshore Profiler Mooring (CE09OSPM; black diamond), and three National Oceanic and Atmospheric Administration (NOAA) National Data Buoy Center (NDBC) buoys 46,029, 46,041, and 46,087 (blue circles). The inset map shows North America with the geographic bounds of the regional map indicated as a blue box. (Right) The CE09OSPM mooring diagram showing the McLane® Moored Profiler, which travels vertically three to four times per day along a jacketed wire rope at approximately 25 cm/s between two bump stops located at 35 and 510 m depth.

The 3244 gridded, daily averaged temperature, practical salinity, potential density, and dissolved oxygen profiles described here are available via Zenodo at https://doi.org/10.5281/zenodo.15627742 [11]. The data set consists of CSV (Comma Separated Values) raw data (A*.TXT, C*.TXT, and E*.TXT) and associated sensor calibration files (*ce09ospm_raw_data.zip*) collected by the OOI Washington Offshore Profiler Mooring (CE09OSPM) located at 46.8517°N, 124.982°W (Fig. 1) between October 2014 and May 2025. Additionally, the data set contains three NetCDF files that follow CF (Climate and Forecast) metadata conventions: *ce09ospm_gridded_profiles.nc* contains observations gridded to a daily, 0.5 dbar pressure grid (Table 1). *ce09ospm_gridded_profile_climatologies.nc* contains daily climatologies, calculated using harmonic analysis over the 10-year period January 2015 to December 2024 for temperature, practical salinity, potential density, and dissolved oxygen. *ce09ospm_gridded_profile_climatology_coefficients.nc* contains the associated three-harmonic linear regression model coefficients for all four variables, which can be used to construct seasonal cycles at temporal resolutions that best fit user needs.

**Table 1:** Names, descriptions, and units of variables included in the *ce09ospm_gridded_profiles.nc and ce09ospm_gridded_profile_climatologies.nc* NetCDF files.

Variable Name	Variable Description	Units
time	Time	days since 1970–01–01 00:00:00 UTC
depth	Seawater depth calculated from pressure	meters
pressure	Seawater pressure	dbar
temperature	Seawater temperature	°C
salinity	Seawater practical salinity	1
potential_density	Potential density of seawater calculated from absolute salinity, potential temperature with respect to a reference seawater pressure of 0 dbar	kg m^-3^
dissolved_oxygen	Seawater dissolved oxygen concentration	micromole kg^-1^
latitude	Latitude	°N
longitude	Longitude	°E

## 4 Experimental Design, Materials and Methods

**Fig. 2:**
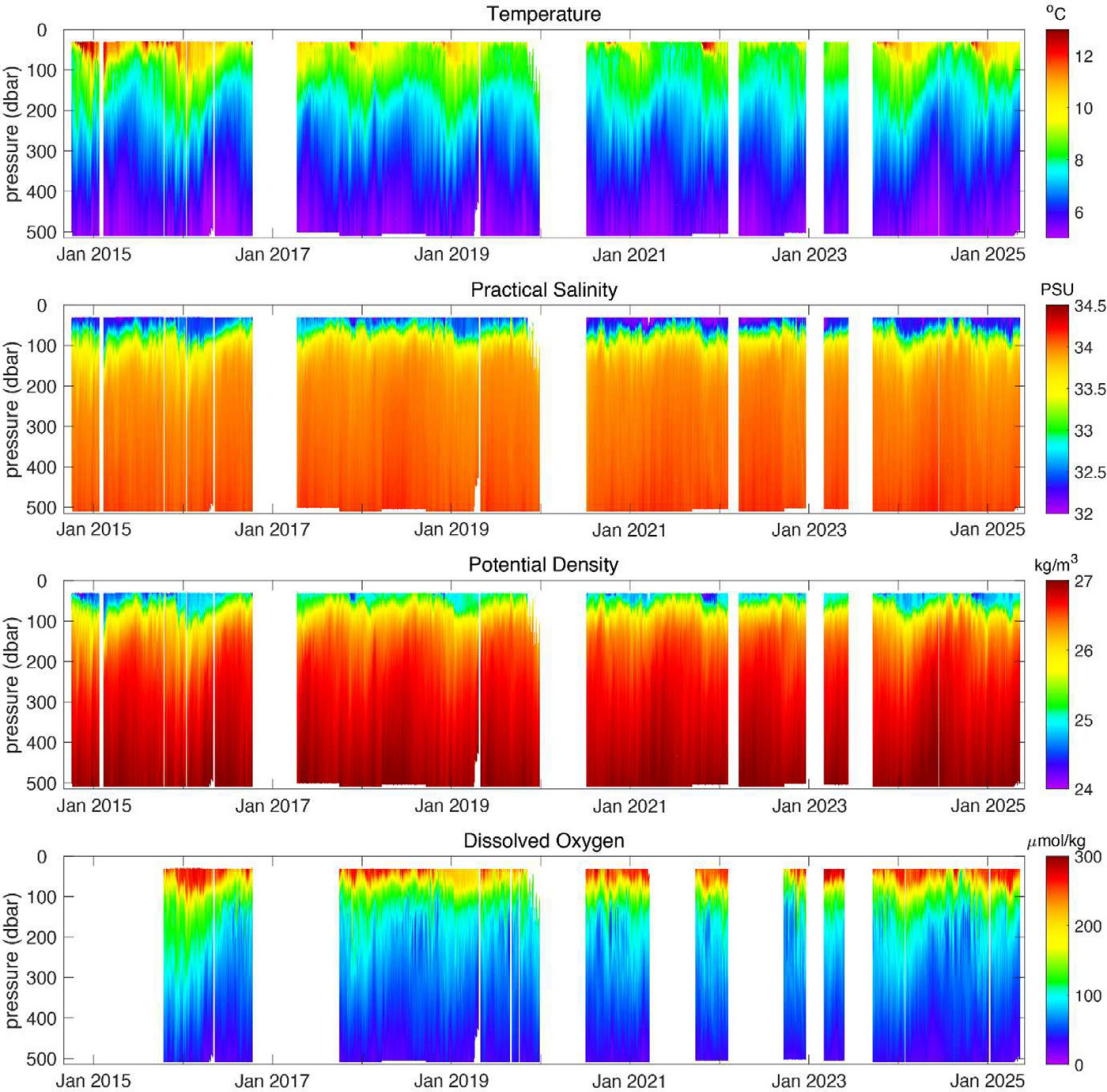
Daily averaged Washington Offshore Profiler Mooring (CE09OSPM) temperature, practical salinity, potential density, and dissolved oxygen (top to bottom panels, respectively) time series from October 2014 – May 2025. The data gap from October 2016 – March 2017 is the result of a buoy mechanical failure during heavy weather. Most other data gaps are the result of either MMP software or hardware failures, or instrument failures.

Raw binary data files [C*.DAT (CTD data); E*.DAT (engineering data plus auxiliary sensor data) and A*.DAT (current meter data)], collected between October 2014 and May 2025 by the Washington Offshore Profiler Mooring, were converted to ASCII text files using the McLane® Research Laboratories, Inc. *Profile Unpacker* v3.10 application (available online at https://mclanelabs.com/profile-unpacker/). Dissolved oxygen calibration files for each of the twenty deployments were downloaded from the Ocean Observatories Initiative asset-management GitHub® repository (available online at https://github.com/oceanobservatories/asset-management). The unpacked C*.TXT (CTD data); E*.TXT (engineering data plus auxiliary sensors) and A*.TXT (current meter data) ASCII data files associated with each deployment were processed using a MATLAB® toolbox that was specifically created to process OOI MMP data. The toolbox, which is available at https://github.com/oceanobservatories/mmp_toolbox, imports MMP A*.TXT, C*.TXT, and E*.TXT data files, and applies the necessary calibration coefficients and data corrections, including adjusting for thermal-lag, flow, and sensor time constant effects. *mmp_toolbox* calculates dissolved oxygen concentration using the methods described in Owens and Millard [12] and Garcia and Gordon [13]. Practical salinity and potential density are derived using the Gibbs-SeaWater Oceanographic Toolbox [14]. After the corrections and calculations for each profile are complete, the data are binned in space to create a final, 0.5-dbar binned data set. The >24,000 individual temperature, practical salinity, pressure, potential density, and dissolved oxygen profiles were temporally averaged to form the data set described here, which consists of 3244 daily mean profiles (Fig. 2).

**Fig. 3:**
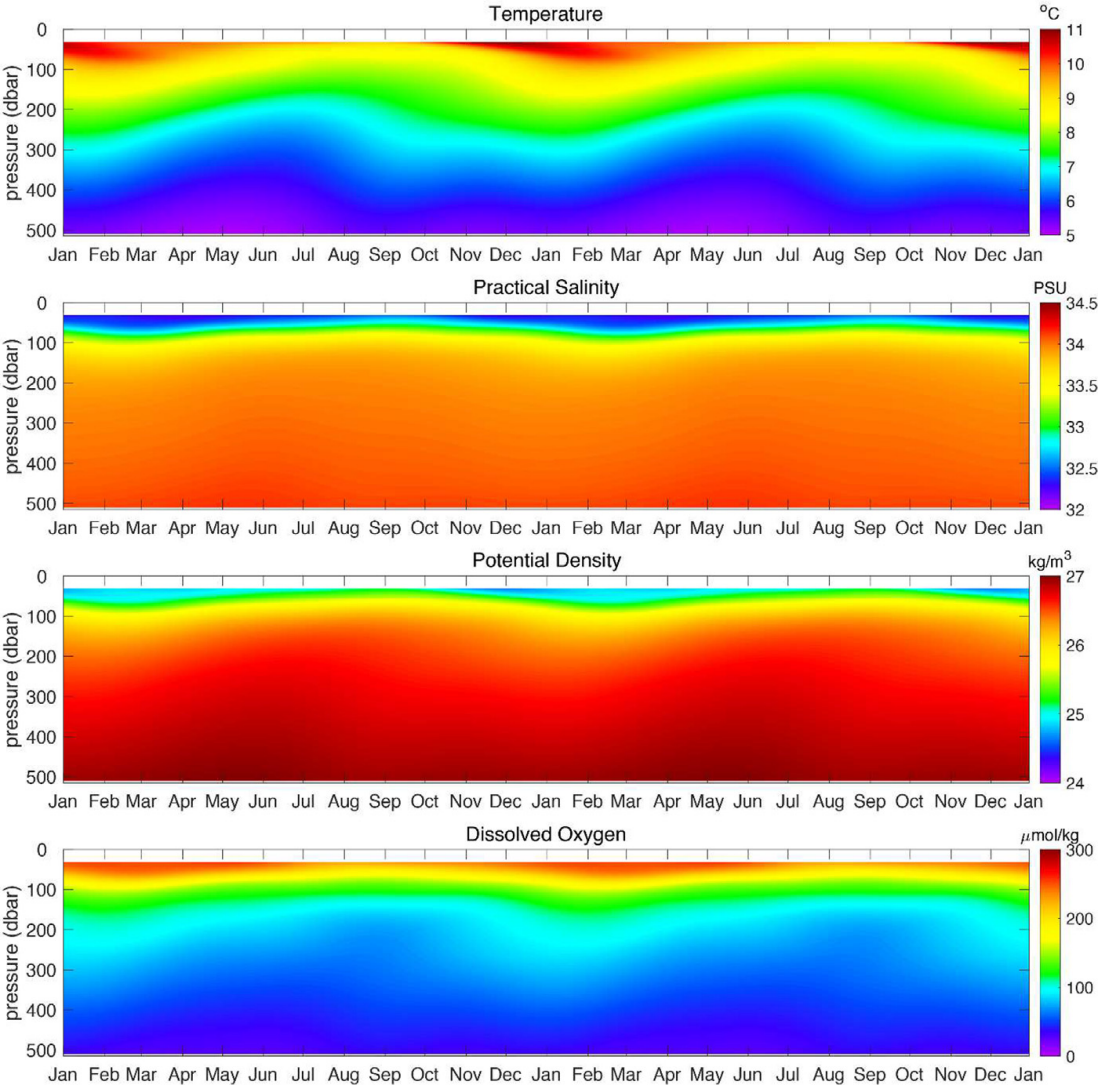
Washington Offshore Profiler Mooring (CE09OSPM) seasonal cycles for temperature, practical salinity, potential density, and dissolved oxygen (top to bottom panels, respectively). The seasonal cycles for each depth bin are based on a least squares fit to the daily averaged, 10-year time series January 2015 to December 2024.

**Fig. 4:**
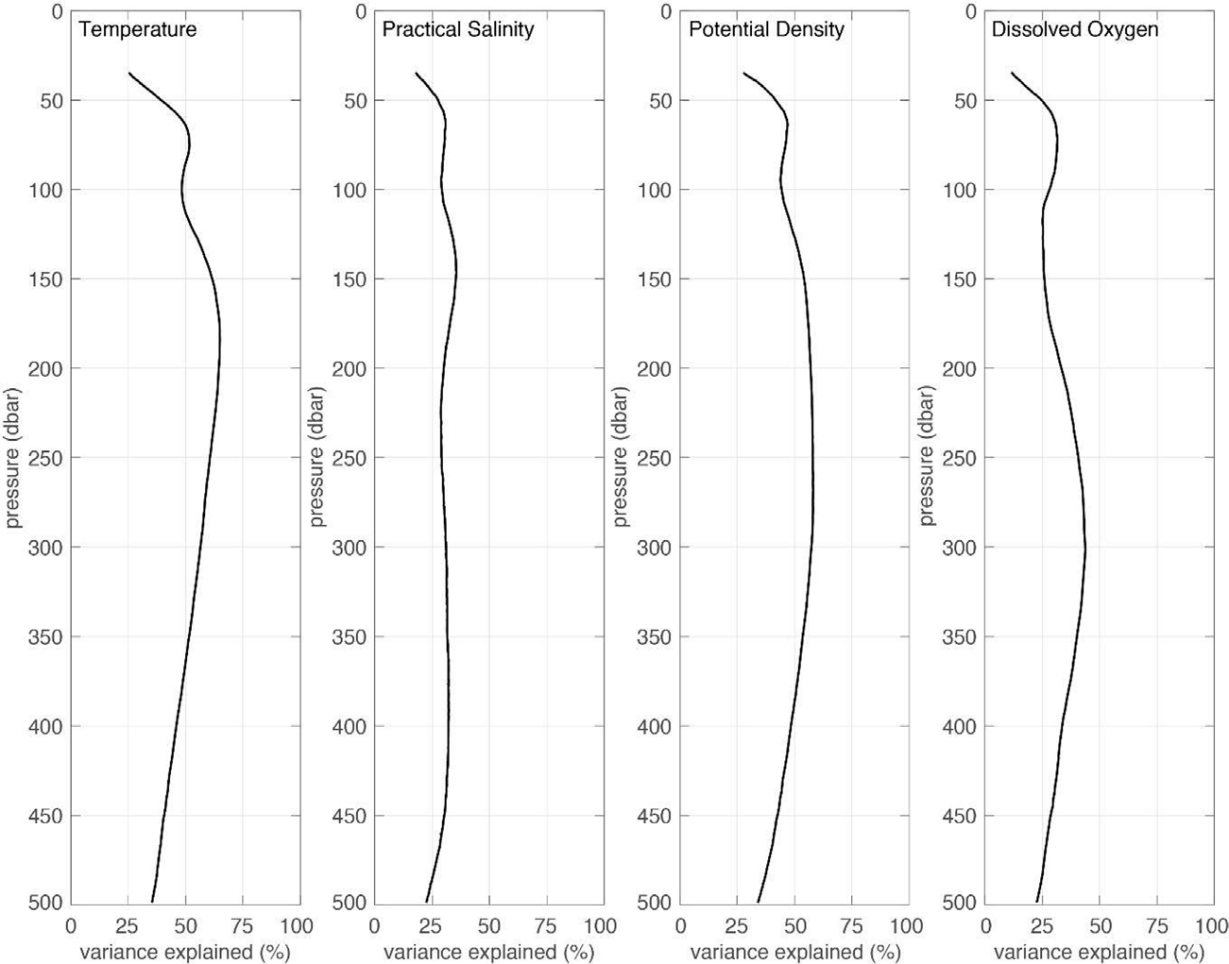
The percent variance explained by the seasonal cycle, calculated as a least squares fit to the daily averaged, 10-year time series January 2015 to December 2024, for each depth bin for temperature, practical salinity, potential density, and dissolved oxygen (left to right panels, respectively).

Using the methods described in Risien et al. [15], daily temperature, practical salinity, potential density, and dissolved oxygen climatologies were calculated for each 0.5-dbar depth bin using a least squares fit of the annual, semi-annual, and triannual harmonics based on the 10-year period January 2015 to December 2024 (Fig. 3). Fig. 4 shows the percent variance explained by the three-harmonic seasonal cycle for temperature, practical salinity, potential density, and dissolved oxygen.

## Limitations

The processing steps described above do not include correcting for sensor drift within a deployment. Sensors were, however, vendor calibrated annually after each mooring recovery. Sea-Bird Scientific estimates that the typical drift rates associated with CTD temperature, conductivity and pressure sensors are 0.0002 °C/month, 0.0003 Siemens/meter/month, and ± 0.05 % full scale range/year, respectively [16]. The calibration drift rate of the SBE-43 is estimated to be <0.5 % over 1000 h of operation [16,17].

## Ethics Statement

The authors have read and adhered to the ethical requirements for publication in Data in Brief and confirm that the current work does not involve human subjects, animal experiments, or any data collected from social media platforms.

## CRediT authorship contribution statement

**Craig M. Risien:** Writing – original draft, Data curation, Validation. **Russell A. Desiderio:** Writing – review & editing, Data curation, Validation. **Jonathan P. Fram:** Supervision, Writing – review & editing. **Edward P. Dever:** Supervision, Writing – review & editing.

## Data Availability

ZenodoGridded Ocean Observatories Initiative Washington Offshore Profiler Mooring (CE09OSPM) Conductivity–Temperature–Depth (CTD) and dissolved oxygen data, 2014 – 2025 (Original data)
